# Allergic Bronchopulmonary Aspergillosis in Trinidad: A Case Report

**DOI:** 10.7759/cureus.19527

**Published:** 2021-11-13

**Authors:** Dominic Dalip, Brandon J Scott, Stanley Giddings, Fidel S Rampersad, Shiva Jaggernauth

**Affiliations:** 1 Internal Medicine, Southern Medical Clinic, San Fernando, TTO; 2 Emergency Medicine, Southern Medical Clinic, San Fernando, TTO; 3 Faculty of Clinical Medical Sciences, The University of the West Indies, Port of Spain, TTO; 4 Interventional Radiology, Southern Medical Clinic, San Fernando, TTO; 5 Pulmonary Medicine, Southern Medical Clinic, San Fernando, TTO; 6 Pulmonary Medicine, Apley Medical Ltd, San Fernando, TTO

**Keywords:** aspergillus fumigatus, asthma, eosinophilia, immunoglobulin e, allergic bronchopulmonary aspergillosis

## Abstract

Allergic bronchopulmonary aspergillosis (ABPA) is an immunologically mediated disease resulting from a hypersensitivity reaction to *Aspergillus fumigatus*. ABPA is identified by bronchial asthma, peripheral eosinophilia, high levels of serum immunoglobulin E, pulmonary infiltration, mucoid impaction, and central bronchiectasis. Diagnosing ABPA is important to consider since there are treatment options that are readily available and response to therapy yields positive results. We describe a case of ABPA present in Trinidad, West Indies, which was not described previously in the literature.

## Introduction

Allergic bronchopulmonary aspergillosis (ABPA) is an immunologically mediated disease resulting from a hypersensitivity reaction to *Aspergillus fumigatus* [1]. The prevalence in adult patients with asthma is estimated to be about 1-2% and 2-15% in patients with cystic fibrosis [2-5]. ABPA is identified by bronchial asthma, high levels of serum immunoglobulin E (IgE), peripheral eosinophilia, pulmonary infiltration, mucoid impaction, and central bronchiectasis [1,6]. There is no single diagnostic test for ABPA and over the years, there have been many proposed criteria, from the Rosenberg-Patterson criteria proposed in 1977 to the International Society for Human and Animal Mycology (ISHAM) working group diagnostic criteria proposed in 2013 [7]. The ISHAM working group proposed that ABPA patients must have a predisposing factor, for example, cystic fibrosis or bronchial asthma. Additionally, elevated Aspergillus-specific IgE antibody levels or immediate cutaneous hypersensitivity to Aspergillus antigens and total IgE levels >1,000 IU/mL must be established [2,7]. Moreover, two of the following must be shown on laboratory testing: radiographic lung infiltrates consistent with ABPA, eosinophilia (>500 cells/μL), or the presence of serum precipitins (immunoglobulin G antibodies to Aspergillus) [6,7]. Patients with ABPA tend to require long-term treatment with corticosteroids and in several cases antifungal agents, potentially leading to several adverse effects. This can be attributed to the fact that ABPA is usually progressive and infrequently remits completely. Upon review of the literature, there were no published case reports on ABPA in Trinidad and Tobago. We describe a case of ABPA presented at Southern Medical Clinic (SMC), Trinidad, West Indies.

## Case presentation

**Figure 1:**
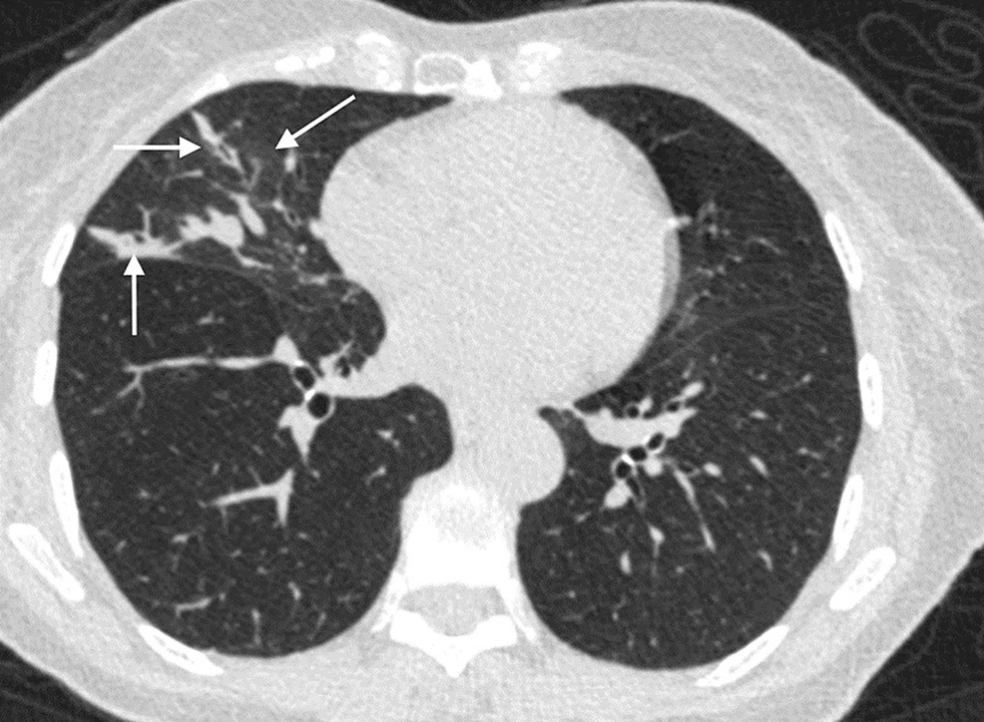
High-resolution CT image through the mid-chest demonstrating ground-glass opacities (oblique arrow), smooth bronchial wall thickening (horizontal arrow), and atelectasis (vertical arrow) within the middle lobe of the right lung.

**Figure 2:**
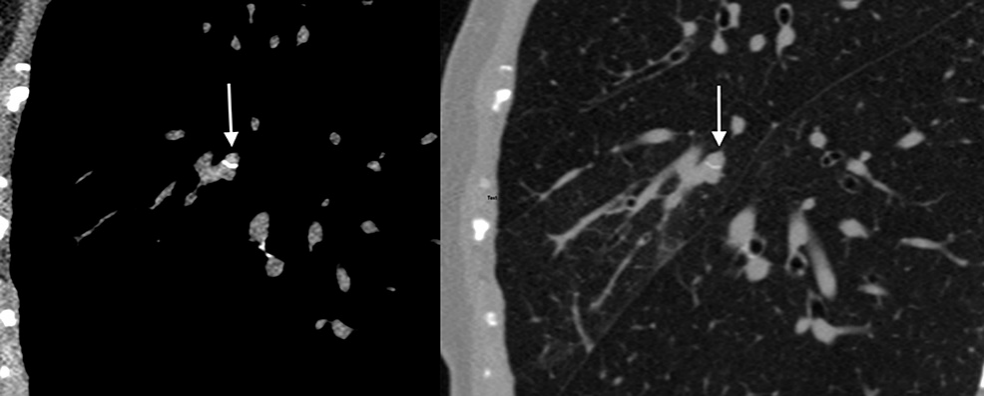
Axial soft tissue window (left) and lung window (right) showing hyperdense mucus plugging (vertical arrow) within the right middle lobe lateral segmental bronchus.

A 73-year-old female with a history of gastroesophageal reflux disease (GERD), sinusitis, hypercholesterolemia, and depression presented to SMC with a cough for the past six months. The cough was non-productive, nocturnal, and associated with occasional wheeze. The patient admitted that she had dyspepsia, allergic rhinitis, and a 15-pound weight loss in the last six months despite a good appetite. The patient denied fever, night sweats, chest pain, exertional dyspnea, hematemesis, nausea, and vomiting. Initial vital signs were unremarkable, and her examination was only notable for fine crepitations at the base of the right lung. Labs on presentation were notable for eosinophilia (2.0; N 0.4-0.6) and an elevated IgE (5,443 IU/ml; N <214 IU/ml). Of note, glycosylated hemoglobin (HbA1c) and thyroid function tests were within normal limits. Chest X-ray (CXR) showed right basilar infiltrates and was otherwise unremarkable. High-resolution computed tomography (HRCT) demonstrated segmental consolidative changes in the right middle lobe (RML), bronchiolar impaction, bronchiectasis, and bronchial wall thickening (Figures 1, 2). No obstruction was noted and lymph nodes measuring <0.5 cm were seen in the hilar region. Sputum samples sent for acid-fast bacilli stain were negative, but gram stain showed pus cells and yeast cells with hyphae and spores that cultured as scanty growth of *Candida albicans*. Pulmonary function test (PFT) showed a restrictive defect with no significant reversibility, and diffusion defect, which corrects for volume seen in Table 1. Diagnoses at this time were asthma and a restrictive lung defect secondary to RML consolidation; this was thought to be due to a fungal infection, most likely Aspergillus. The patient was optimized on oral prednisolone and anticholinergics and then referred to the infectious disease team for further evaluation. To confirm the diagnosis of ABPA, the Aspergillus IgE antibody test was conducted, and results deduced a positive result. A two-week peak flow diary reading was performed and reviewed, which showed variability of 25%, consistent with asthma, although reversibility was not demonstrated in the PFT. Moreover, the patient had a clinical response to steroids and bronchodilators. The diagnosis of asthma was then established, which is fundamental to the diagnosis of ABPA.

**Table 1:** Pulmonary function test showing restrictive effect without significant reversibility using salbutamol as the bronchodilator agent. Pred, predicted; LLN, lower limit of normal; Chg, change; Abs, absolute; FVC, forced vital capacity; FEV, forced expiratory volume; FEF, forced expiratory flow; PEFR, peak expiratory flow rate; FIF, forced inspiratory flow; MVV, maximum voluntary ventilation; TLC, total lung capacity; VC, vital capacity; RV, residual volume; FRC, functional residual capacity; SVC, slow vital capacity; IC, inspiratory capacity; ERV, expiratory reserve volume; DLCO, diffusion capacity of lung for carbon monoxide; VA, alveolar ventilation.

Spirometry	Pred	LLN	BEST	% Pred	BEST	% Pred	% Chg	Abs
FVC (L)	2.20	1.52	1.38	63	1.42	65	3%	40 mL
FEV1 (L)	1.69	1.13	0.99	59	1.07	63	8%	80 mL
FEV1/FVC (%)	78	69	72	92	75	96	4%	
FEV6 (L)			1.3		1.36		5%	60 mL
FEF25-75 [iso] (L/s)	1.63	0.27	0.66	40	0.98	60	48%	320 mL
PEFR (L/s)	4.79	1.94	3.62	76	3.87	81	7%	0.25 L/s
FEF50 (L/s)	1.31		1.04	79	1.72	131	65%	680 mL
FIF50 (L/s)			0.31		1.81		484%	1500 mL
FEF50/FIF50			3.35		0.95		-72%	-2400 mL
MVV (L/m)	73.9	72.9						
Lung volumes								
TLC (L)	4.20	3.14	2.63	63				
VC (L)	2.20	1.52	1.38	63				
RV (L)	2.00	1.23	1.25	62				
RV/TLC (%)	45	34	48	107				
FRC (L)	2.44	1.38	1.77	73				
SVC (L)	2.20	1.52						
IC (L)	1.76	0.70	0.86	40				
ERV (L)	0.44		0.52	118				
Diffusing capacity								
DLCO (ml/min/mmHg)	13.00	7.00	7.92	61				
DLCO [Hb] ml/min/mmHg	13.00	7.00	8.24	63				
DLCO/VA (ml/min/mmHg)	3.55	1.71	3.73	105				
VA [BTPS] (L)	4.31	4.23	2.21	51				

The patient was followed up in three months with repeat HRCT and PFT. The HRCT showed that the consolidative changes within the middle lobe of the right lung have resolved, with only minimal atelectasis seen. There was minimal bronchiectasis and mucus plug seen, with the middle lobe of the right lung laterally. Stable atelectasis was noted in the left lingula. The lungs and pleural spaces are otherwise normal. The PFT also showed significant improvement as demonstrated in Table 2.

**Table 2:** Repeat pulmonary function test demonstrating improvement using salbutamol as the bronchodilator agent. Pred, predicted; LLN, lower limit of normal; Chg, change; Abs, absolute; FVC, forced vital capacity; FEV, forced expiratory volume; FEF, forced expiratory flow; PEFR, peak expiratory flow rate; FIF, forced inspiratory flow; MVV, maximum voluntary ventilation; TLC, total lung capacity; VC, vital capacity; RV, residual volume; FRC, functional residual capacity; SVC, slow vital capacity; IC, inspiratory capacity; ERV, expiratory reserve volume; DLCO, diffusion capacity of lung for carbon monoxide; VA, alveolar ventilation.

Spirometry	Pred	LLN	BEST	% Pred	BEST	% Pred	% Chg	Abs
FVC (L)	2.20	1.52	1.70	77	1.73	79	2%	30 mL
FEV1 (L)	1.69	1.13	1.27	75	1.29	76	2%	20 mL
FEV1/FV (%)	78	69	75	96	75	96	0%	
FEV6 (L)			1.65		1.65		0%	
FEF25-75 [iso] (L/s)	1.63	0.27	1.06	65	1.08	66	2%	20 mL
PEFR (L/s)	4.79	1.94	4.97	104	4.93	103	-1%	-0.04 L/s
FEF50 (L/s)	1.31		1.44	110	1.71	131	19%	270 mL
FIF50 (L/s)			0.32		0.33		3%	10 mL
FEF50/FIF50			4.50		5.18		15%	680 mL
MVV (L/m)	73.9	72.9						
Lung volumes								
TLC (L)	4.20	3.14	3.01	72				
VC (L)	2.20	1.52	1.70	77				
RV (L)	2.00	1.23	1.31	66				
RV/TLC (%)	45	34	44	98				
FRC (L)	2.44	1.38	1.91	78				
SVC (L)	2.20	1.52						
IC (L)	1.76	0.7	1.10	62				
ERV (L)	0.44		0.60	136				
Diffusing capacity								
DLCO (ml/min/mmHg)	12.63	6.63	11.24	89				
DLCO [Hb] ml/min/mmHg	12.63	6.63	11.58	92				
DLCO/VA (ml/min/mmHg)	3.55	1.71	4.35	123				
VA [BTPS] (L)	4.31	4.23	2.66	62				

## Discussion

It is desirable to get an early diagnosis of ABPA, as disease progression can result in complications such as cavitary disease or end-stage fibrosis, bronchiectasis, pulmonary hypertension, and respiratory failure. The differential diagnosis for ABPA includes Aspergillus-sensitive asthma without allergic bronchopulmonary Aspergillus, allergic bronchopulmonary mycosis, refractory asthma, chronic obstructive pulmonary disease, bronchiectasis (due to other causes), pulmonary tuberculosis, eosinophilic pneumonia (acute or chronic), eosinophilic granulomatosis with polyangiitis, and hyperimmunoglobulin E syndrome, among others. It is important to make an accurate diagnosis as management varies significantly based on the condition diagnosed. However, the correct choice of investigations to make an accurate diagnosis depends on the following two factors: the prevalence of the disease and the criteria used to make the diagnosis [8,9].

There are multiple sets of diagnostic criteria for ABPA, and these include the Rosen-Patterson criteria, ABPA in cystic fibrosis, minimum essential criteria, minimum criteria and additional criteria, truly minimal criteria as well as ISHAM working group criteria [7,10].

The Rosen-Patterson criteria, which were only used for patients with asthma, had eight major and three minor criteria. However, there was controversy on the number of criteria that were needed to make the diagnosis, and cut-off values for some of the lab tests were not specified [7]. Over the years, additional criteria were developed to include patients with cystic fibrosis as well as develop minimum criteria needed to make the diagnosis. The ISHAM working group later proposed a new set of criteria for the diagnosis of ABPA, which was published in 2013.

It can be challenging for the clinician to establish the diagnosis of ABPA with full confidence. There is no single diagnostic test and hence the clinician relies upon the medical literature and these sets of diagnostic criteria. As these criteria are applied, data have emerged comparing some of the individual tests as examined further. Of note, diagnostic imprecision is sure to diminish with the exclusion of serum eosinophil and precipitin levels as part of the criteria since they were neither specific nor sensitive for a diagnosis of ABPA established in a previous large cohort of patients [10,11]. However, it is used to support the diagnosis and together with other positive findings can assist in confirming the diagnosis. Aspergillus specific IgE antibody was more specific than Aspergillus skin test, which can have issues related to the quality of antigen and competence related to the technical performance of the test [2,4].

In our case, both Aspergillus-specific IgE antibody test was positive and serum eosinophil count was elevated. This gave us sufficient evidence to consider the diagnosis of ABPA in this case. In theory, chest radiography may be used to determine which patients should undergo CT scanning, with the added advantage of detecting pulmonary infiltrates [8,12]. The CXR in our patient demonstrated right basilar infiltrates in keeping with our suspected diagnosis. In HRCT, central bronchiectasis combined with centrilobular nodules and mucus impaction (especially high attenuation mucus) strongly favor the diagnosis of ABPA [13]. Other bronchial abnormalities include dilated and totally occluded bronchi, tubular opacities and dense circular opacities, bronchial wall thickening, air-fluid levels within dilated bronchi, and parallel-line opacities extending to the periphery [14]. The common parenchymal abnormalities seen were nonhomogeneous patchy consolidation and parenchymal scarring of varying extent [14]. HRCT in our patient demonstrated segmental consolidative changes in the RML, bronchiolar impaction, bronchiectasis, and bronchial wall thickening. No obstruction was noted and <0.5 cm lymph nodes were seen in the hilar region. These changes on HRCT are consistent with the diagnosis of ABPA. These patients would usually be stage as ABPA-B (ABPA with bronchiectasis). However, findings of bronchiectasis on CT chest are not necessary for the diagnosis as patients can be staged as ABPA-S (ABPA serological), ABPA-HAM (ABPA with high attenuation mucus), and ABPA- CPF (ABPA with pleuropulmonary fibrosis).

The use of antifungals is still debatable in the treatment of ABPA. It was presumed that there would be a reduction in antigenic stimulation by decreasing the fungal load and thus a decline in the inflammatory response. Patients on long-term oral prednisolone have traditionally switched to itraconazole as a corticosteroid-sparing agent [14]. In our case, we started our patient on oral corticosteroids, which she responded well to. If the patient still had persisting symptoms despite steroids therapy, itraconazole would have been initiated.

## Conclusions

In conclusion, we report a case of ABPA in Trinidad, West Indies that was not previously reported in the literature. The patient met the diagnostic criteria of ABPA with supporting evidence of Aspergillus-specific IgE antibody positivity and serum eosinophilia. HRCT demonstrated central bronchiectasis combined with centrilobular nodules and mucus impaction (especially high attenuation mucus), strongly favoring the diagnosis of ABPA. Upon a three-month follow-up, the patient showed significant improvement on corticosteroids without the need for itraconazole therapy. We hope that this case report may raise awareness for the diagnosis and management of ABPA in Trinidad and will encourage other physicians to publish more cases to establish optimal medical therapy and possible management in treatment-resistance cases.
